# Family Communication about End-of-Life Decisions and the Enactment of the Decision-Maker Role

**DOI:** 10.3390/bs7020036

**Published:** 2017-06-07

**Authors:** April R. Trees, Jennifer E. Ohs, Meghan C. Murray

**Affiliations:** Department of Communication, Saint Louis University, 3733 West Pine Blvd, Xavier 300, St. Louis, MO 63108, USA; johs@slu.edu (J.E.O.); mmurra28@slu.edu (M.C.M.)

**Keywords:** end-of-life decision-making, family roles, surrogate decision maker

## Abstract

End-of-life (EOL) decisions in families are complex and emotional sites of family interaction necessitating family members coordinate roles in the EOL decision-making process. How family members in the United States enact the decision-maker role in EOL decision situations was examined through in-depth interviews with 22 individuals who participated in EOL decision-making for a family member. A number of themes emerged from the data with regard to the enactment of the decision-maker role. Families varied in how decision makers enacted the role in relation to collective family input, with consulting, informing and collaborating as different patterns of behavior. Formal family roles along with gender- and age-based roles shaped who took on the decision-maker role. Additionally, both family members and medical professionals facilitated or undermined the decision-maker’s role enactment. Understanding the structure and enactment of the decision-maker role in family interaction provides insight into how individuals and/or family members perform the decision-making role within a cultural context that values autonomy and self-determination in combination with collective family action in EOL decision-making.

## 1. Introduction

In the United States, when an individual is incapacitated at the end of life and cannot make a decision for him or herself, family members often are called upon to make decisions for the individual. End-of-life (EOL) care decisions encompass decisions to initiate, withhold, continue, or end life-sustaining treatment. Making a decision for a family member at the end of life is one of the most emotionally difficult decisions families will ever face [1,2]. How this decision is managed has important implications for the patient’s quality of life at the end [3] and affects family members’ emotional well-being long after the decision has been made [1,2]. During decision-making, families encounter various dilemmas and challenges, including uncertainty about what to do and how to behave [4]. 

As they navigate this emotionally-charged experience and coordinate action together, family members take on roles in the decision-making process that may be supported or challenged by others in the interaction. One particularly important role in this context is the decision-maker role itself [5]. The U.S. legal and medical systems encourage the use of an advance care directive (ACD) to designate a formal decision maker, although many individuals do not engage in EOL planning [6]. In contrast to other legal systems (e.g., the United Kingdom where clinicians serve as default decision makers based on best interests), in the U.S., a family member typically takes on the decision-maker role in cases where a patient can not make decisions for him or herself, using substituted judgment or best interests to guide the decision-making [7,8]. The decision-maker role, however, is rarely enacted in isolation. Multiple family members, for example, often participate in the decision-making interaction [9,10]. Given the interdependence of the family and the value of family engagement in the decision-making process, expectations and behaviors for the decision-maker role likely emerge in and are shaped by interaction with others in the family. Understanding the enactment of the decision-making role in family interactions about EOL decisions provides insight into how family members coordinate EOL interaction together and either support or undermine the performance of the decision-maker role.

### 1.1. Family Communication and End-of-Life Decision-Making

In the U.S., the legal and medical systems emphasize individual autonomy in health-care decision-making, expecting decision makers to follow the wishes of the individual [11]. Individuals are encouraged to engage in EOL planning and complete formal documentation that specifies preferences regarding life-sustaining treatment and names a surrogate decision maker [12]. Despite this emphasis on advance care planning, the percentage of U.S. adults completing an advance care directive (ACD) is relatively low [6]. Several different surveys have demonstrated that many individuals feel that talking to family members about EOL wishes and having written documentation of wishes are both important, but a low percentage of respondents have actually had conversations with family and even fewer have legal documentation in place [13,14]. 

Most often, in situations where a surrogate decision maker has been established, the individual identified as a surrogate is a family member (e.g., spouse, child or grandchild) [15,16]. This reflects a dominant preference across cultural groups in the United States for family to be involved in surrogate decision-making [3,17,18]. Additionally, regardless of whether or not a formal decision maker has been identified in an ACD, multiple family members usually participate in decision-making conversations when a decision must be made [9,10,19], and some families may expect consensus in the decision [20]. 

The complexity of EOL decision situations creates a number of dilemmas for family members required to make a decision for a loved one at the end of life. Family members may face challenges in obtaining the information needed to make a good decision [21] or knowing when a decision point is nearing [22]. Without adequate information from health care providers to inform the decision, families can experience resentment and emotional burden after a decision is made [21,22]. Family members also may be uncertain about the right decision to make [10], even when there is an ACD in place [4]. Living wills, for example, do not always provide insight into the specific decision that must be made [23]. Additionally, when family members are aware of the patient’s wishes, they still may encounter a contradiction between their own desires and the patients’ desires [24], often experienced as a tension between holding on and letting go [25]. Family members also may struggle to make sense of the decision and of a loved one’s likely death. In interaction during clinician-family conferences in an intensive care unit, for example, family members grappled with understanding withdrawing or withholding life support as killing a loved one versus seeing it as letting him or her die [24]. They also experienced a contradiction between perceiving death as a burden or as a benefit. 

As family members make decisions, they can experience challenges in effectively coordinating family decision-making and working with medical professionals. Families, for example, sometimes experience conflict and disagreement as multiple family members participate in the decision-making process. Family members may disagree over who should be included in the decision-making [24] or, ultimately, what decision to make [5]. A history of family conflict prior to the EOL decision, communication in which family members try to assert control over the decision, and families having difficulty talking with each other about the situation all predict greater family conflict when making an EOL decision [26]. Family conflict in EOL decision-making can lead to more aggressive treatment [27] and reduce the degree to which decisions match the preferences of the patient [28]. Conflict in the family during decision-making also may undermine the quality of family relationships after a decision has been made [29]. 

### 1.2. Family Roles and End-of-Life Decision-Making

Clearly family interaction around EOL decisions is fraught with complications that affect the well-being of both the patient and the family. As families negotiate this unfamiliar communicative terrain, members take on a variety of informal roles in the decision-making process [5]. Roles refer to communicatively negotiated understandings of behavior to be enacted by family members in particular positions [30,31]. Roles are communicatively created and recreated in interaction with others [30]. Roles both develop in and then guide interaction as family members form expectations about how someone holding a particular role will act. As a family faces an EOL decision, family members in both formal (e.g., designated surrogate decision maker) and informal decision-making roles must work out what their roles mean for family communication and decision-making behaviors. 

Within family interaction, the behaviors of others shape how a role is enacted. Role appropriation is shaped by role expectations and others in the family can facilitate or undermine the enactment of a particular role, which may need to be negotiated among family members [32]. Additionally, Salazar [31] recognizes that roles that develop in groups are shaped by both past and present interaction and have environmental constraints. In EOL decision-making, roles likely build on past family interactional histories, formal familial roles (e.g., spouse, child), and gender role expectations [30,33], but also adapt to the unique decision-making situation facing the family. The larger medical and legal systems in the U.S. also add an extra layer of expectations for formal role assignments that shape interaction within the family and between the family and medical professionals [21]. Although there might be a variety of different informal roles that develop in the family system when making an EOL decision for a loved one [5], of particular interest for this project is the decision-making role itself. More specifically, we pose the following research question:

RQ: How do family members enact the decision-making role in EOL decision-making interactions?

## 2. Materials and Methods

### 2.1. Participants

Individuals who participated in family decision-making for a loved one at the end of life, either as a surrogate decision maker or as a family member involved in the communication about the decision, were interviewed about their family communication during the decision-making process. Participants were recruited via an announcement in a University newsletter, announcements on Facebook, researchers’ social networks, and referrals from participants. We interviewed 22 participants, 19 women and 3 men. Participants had an average age of 44 (range = 18 to 70). Fourteen participants identified as Caucasian, four as African American, two as Asian, one as Dominican, and one as Indian. Eight participants indicated that their family member at the end of life had formally designated a surrogate decision maker. Seven participants reported that their family member had not designated a surrogate, and six were unsure. One person reported on a decision involving a family member who did not reside in the United States at the end of life and was unsure of legal means and options to designate a surrogate decision maker for the person at the end of life.

Many of the decisions faced by families in this study involved decisions about initiating or withdrawing life support. Others included decisions about pursuing specific interventions (e.g., surgery, chemotherapy), stopping treatment and going on hospice, and whether or not a patient could return home or must remain in the hospital. In many cases, decision makers and families faced a number of different decisions as they navigated the end of life with their loved one. All of the decisions except one were made within the context of the health-care and legal system in the United States. However, decision narratives involved families of mixed citizenships and cultures. In one case, the family member at the end of life had moved from her lifelong residence in Singapore to the United States to be with her family during her final days. Thus, the experiences reported here represent a variety of cultural and familial backgrounds that are influenced by the communication surrounding EOL care within the U.S. medical and legal system. 

Three participants had made more than one EOL decision on behalf of a family member, yielding 25 different decision situations in the data. In two cases, family members represented a patient who was not entirely incapacitated. Decisions were made for parents (15), siblings (4), grandparents (3), a mother-in-law (1), an aunt (1), and a nephew who was fictive kin (1). Respondents reported on decisions that were made between 1 and 10 years prior to the interview, with an average time between the decision and the interview being 3.75 years. Respondents reported on various roles in the decision. Eight individuals reported that they were one of the persons who made the EOL decision in the family. Six reported that they were the sole surrogate decision maker. Three people indicated they were consulted about the decision, three witnessed the process of decision-making, and one was told about the decision after it was made. One person indicated that her role was that of the spokesperson for the family.

### 2.2. Procedures and Analysis 

The Saint Louis University Institutional Review Board approved the study. Prior to the interview, participants were asked a series of questions to gather demographic information and information about the nature of the decision-making situation. Interviews were conducted either face-to-face or over the phone and were audio-recorded. The interview protocol invited participants to tell the story of their family communication surrounding a care decision made on behalf of a loved one at the end of life, starting at the point that they became aware that an EOL decision might need to be made. 

The research team consisted of four individuals: three Caucasian women and one Indian-American woman. Interviewers identified as young or middle-aged adults. The two primary investigators trained the other members of the research team to follow the semi-structured interview protocol. Interviews proceeded as a guided conversation, asking a variety of questions about family communication during the process, including what was talked about or avoided, who interacted with whom in what contexts, and how decisions were made. Interviewers were prepared for the emotional nature of the interviews and were attentive to cues of respondent distress. If a respondent became distressed, interviewers were prepared to offer resources and professional referrals for care. Interviews ranged in length from 22 to 94 min. After interviews were completed, they were transcribed, and participants and family members referenced in the interview were given pseudonyms to protect their identities. 

To analyze the interview data, the second two authors independently reviewed each transcript, specifically looking for places in the data where participants talked about the behaviors and expectations associated with the decision-making role in their family interaction and how others supported or challenged that role. Honoring an emic perspective focused on the point of view of interviewees, the authors engaged in open-coding, identifying common patterns and themes in the data related to enactment of decision-making roles. All three researchers then met to compare and contrast the emerging themes, determine points of overlap and similarity and to reconcile differences between the two analyses. After identifying the relevant themes in the data, the second two authors also engaged in axial coding, noting the relationships among the different themes. 

After arriving at a preliminary set of themes, the three authors met again to engage in investigator triangulation [34]. They discussed points of commonality and reconciled differences, finding that they had converged in their analysis. They then read through the transcripts to identify additional themes or negative cases. No new themes emerged and given that the properties and dimensions of each theme were well-developed and that the relationships among the categories were well-established, theoretical saturation was concluded [35]. Two key areas of analysis emerged in this thematic analysis of the interview transcripts, with subthemes within each area that help to develop a clearer understanding of how EOL decision-making roles are enacted in the family. 

## 3. Results

### 3.1. The Structure of Decision-Making Roles within the Family System

When making EOL decisions for a loved one, one or more family members took on the decision-making role. Three different patterns of role enactment were identified in the interview data. The first two patterns of consulting and informing emerged in families with a single designated decision maker who received collective family input. Within these patterns of role enactment, family and gender roles shaped decisions regarding who took on decision-making roles. The third pattern of collaborating emerged in families in which the collective family unit took on the decision-maker role. 

#### 3.1.1. Single Designated Decision Maker with Collective Family Input: Consulting or Informing

In many cases, one individual was designated, formally or informally, as the official decision maker for a loved one’s end-of-life care, but there was also collective family involvement. In these cases, there was a clear expectation that a specific individual would make the decision for the loved one. For a number of families, this role was established by formal legal documentation. Phyllis, for example, had health care power of attorney for her mother and noted, “Throughout the whole decision-making process, obviously, my dad and my sister, we kind of always conferenced as a family, but I was the one making the ultimate decisions on her end-of-life care.” 

In other families, a decision-maker was not specified through legal documents, but family members understood who should make the decision based on legal and cultural understandings of family roles (e.g., spouse rather than children) as well as gender role expectations. For example, Laura indicated that in her Dominican family “it is usually the elder daughter who always makes the decision, but she does not make the decision on her own.” Age also emerged as a factor shaping who took on the decision-making role in the absence of formal documentation. Sheila, for example, noted that “the younger people deferred all of the decision-making to the older people.” 

Less commonly, the decision-maker role emerged through decisive behaviors on the part of a family member in the absence of formal, legal documentation. Beatrice, for example, observed that her mother “wasn’t prepared for the situation because it had occurred so quickly.” Her mother and her brother were in denial about her father’s end of life, and when Beatrice arrived, she took over, asking questions and making arrangements. According to her, “I protected him; I mean I literally felt like his guard dog…. if I hadn’t had been there they would have just kept treating him. Even though there was no treatment.” 

Family roles (e.g., spouse, child), gender roles, and age all emerged as existing roles in the family that shaped expectations regarding who would take on the decision-making role. Specifically, if a spouse was living, he or she was expected to take on the decision-making role, and participants provided explanations for why that did not happen in situations where a child or sibling took on the role instead. The need to protect the husband or wife given his or her emotional distress often emerged as an explanation for role enactment by a child instead. Beatrice, for example, indicated, “My mother, God bless her soul, she was just a bystander…. She could not communicate well with the doctors or the nurses, you know, she just… it was almost as if she was in shock.” Although the gendered nature of the role varied from family to family, descriptions of who should take on decision-making roles intersected with gendered expectations regarding children’s influence and support. Expectations regarding age and family roles were apparent in the data as well. Beatrice, for example, specifically noted that her role in the decision-making was unexpected to her because she was the youngest child and female. In situations where the decision-maker role was held by someone unanticipated (e.g., the youngest sibling), participants typically rationalized the role. For example, some participants pointed to the expertise of the chosen individual (e.g., “I’m the nurse so everyone turned and looked to me,” Sheila), closeness and trust in the relationship with the patient, or their willingness to make difficult decisions. Jackie, for example, said that her mom chose her to be the decision maker even though she was the youngest “because the rest of my siblings were all sissies. She chose the tough one.” 

Regardless of the formality of the decision-maker role, collective family input was an important part of the decision-making process. How individuals enacted the decision-maker role in relationship to this collective input, however, varied across situations. In some cases, like Phyllis’s described above, family conferences and other types of group interaction offered an opportunity for a number of different family members to have input into and, at times, get on board with the decisions that were going to be made. The behaviors of the decision maker involved consulting other family members as a way to enact the decision-maker role. Caroline, for example, talked about her sisters offering different suggestions to her dad about what to do, which he took into consideration as he made decisions for her mom. John’s brother-in-law included his wife’s family in conversations about his wife’s EOL decisions prior to making decisions. In these types of role enactments, the individual in the formal decision-making role integrated the input of family members into the decisions made.

In other cases, the decision-maker role in family interaction involved informing more than consulting. Both listening and explaining were a part of the behaviors enacted in this type of decision-maker role. For example, individuals in the decision-maker role used family interactions to create a space for family members to express themselves before the decision maker made the decision he or she already knew would honor the loved one’s wishes. This offered an opportunity to recognize and hear other family members’ thoughts and opinions. Jackie, for example, said “I would tell them, ‘Hey guys, this is where we are, this is the outlook, this is what things are.’ And it was always… the discussion was always… we’ll do everything possible to keep her alive. And which… I had to always… had to hear them out and allow them to express themselves and allow them to say what their desires were.” Even though Jackie was officially recognized in her mother’s medical record as the surrogate decision maker and knew that her mother should be taken off of life support, she included her family in a collective discussion to give them an opportunity to share before telling them what needed to happen. 

At times, the decision maker used collective interaction as an opportunity to inform the family members of what the loved one would want and explain what the decision should be. Amy, for example, described her mom telling her adult children “this is what’s going on, this is what the best decision was for him, and him and I talked about it before” at their family conference around the kitchen table. When her father had a stroke, Maria said that “my mother looked to me and my son… she said ‘no that is not what we had discussed…. you know, your father and I already discussed it.’” These conversations offered an opportunity for the decision maker to demonstrate knowledge of what was best and provide an argument for it grounded in the desires of the patient.

#### 3.1.2. The Collective Family Unit as Joint Decision Makers: Collaborating

Although most families indicated that one person specifically took on the decision-maker role, a few families did not display this type of role enactment. Instead, family members jointly took on the decision-maker role together as a unit and collaborated together. In the families where this happened, there was a designated surrogate decision maker or a spouse who might have been expected to take on the decision-making role given the formal expectations of the legal and medical systems. That person, however, preferred to structure the interaction around collaborative group decision-making rather than take on the decision-making role individually. Catherine’s mom, for example, had given her sister medical power of attorney. All seven siblings, however, regularly consulted one another as a group on decisions related to her mother’s Alzheimer’s. “It was always really understood that unless we were all on board, we weren’t doing it… So it was all or nothing. Like we weren’t just going to leave one person feeling guilty about something.” Similarly, although Molly’s mother had power of attorney to make decisions for her father after her father’s stroke, Molly’s family had “an official family meeting” around the kitchen table at which her brother read her father’s living will. Each family member then expressed their opinion about life support decisions that needed to be made. Molly noted that even her brother-in-law, whom she had originally felt should have no say, had proven himself to be a strong support and was asked to participate and voice his opinion. In contrast, Teresa’s sister had no advance care directive in place. Her sister’s husband was reluctant to take the decision-maker role on by himself, however, although Teresa noted that “we respected the fact that Justin was her husband and he had the final say.” At one point, he called Teresa and said, “the kids and I want you to come up and help us make a decision about Alice.” Although she initially indicated reluctance to be a part of the decision-making, Teresa, Justin, and her sister’s three children had a family meeting and discussed extensively what they all thought Alice would want and made a decision as a group. 

In these family situations, there was no clear surrogate decision-maker role given to a specific family member in the interaction. Family members were called upon to help make a decision together as a unit rather than one person taking on the decision-maker role alone. Coming together to discuss the loved one’s wishes and focusing on the “we” aspect of the family emphasized the collective role the family enacted. 

### 3.2. Facilitating and Undermining the Enactment of the Decision-Maker Role

Regardless of whether a single decision-maker was designated or emerged during family interactions or the family collectively made a decision, the communication choices of others either facilitated or challenged the decision-maker’s role enactment. Both other family members’ response to the role and the behavior of physicians and medical professionals made a difference in how the decision-maker role was enacted.

#### 3.2.1. Family Members and Role Enactment

Given the importance of family interaction for the decision-making process, it is not surprising that family members’ responses shaped the decision-maker role. This primarily occurred through two means. First, family members sometimes offered supportive behavior that bolstered the decision-maker’s influence and helped him or her cope with the decision to be made. Alternately, conflictual behavior that challenged the decision maker’s right to make the decision or the wisdom of his or her decision-making behavior was also evident. 

One way in which families facilitated decision-making roles was by supporting one another’s suggestions. Agreeing with the decision reinforced the decision maker’s rights and responsibilities in that role. Family support was particularly important for decision makers who took on the role through decisive behavior. Sheila’s sister did not have a surrogate decision maker, and Sheila reported that, “when I told them that enough is enough, don’t put her through surgery, my other sister, she was supportive of that. And then my mom was kind of supportive of that. So, it was really us trying to tell the rest of the family that this is probably the best.” Others’ acceptance of that decision contributed to Sheila’s enactment of the decision-maker role in the absence of legal documentation and/or a family role that would position her as next-of-kin. Support also became important in the context of family conflict. Charlotte’s sister-in-law, for example, did not approve of the choices that her brother-in-law was making for her sister and was calling people in the family about it. Charlotte said everybody else in the family “told her basically we trust Adam’s choices.” Showing support could be seen as a sign of collective solidarity reinforcing the decision-maker role. 

Additionally, family communication also provided an opportunity for other family members to reinforce the decision-maker’s formal role by reminding the designated surrogate decision maker about the EOL care that had already been discussed or the wishes of the patient. Phyllis, for example, noted that “I just had to be the one to sign the paper. And I was always a little hesitant. Umm… And my dad would just kind of chime in and say, ‘You know, remember what you and your mom had discussed last week. Remember what the three of us talked about the week before.’” Edna talked about sitting with her best friend Patty, whose son had been in a car accident. At one point, Patty was talking about how she could not let her son go, and she said, “I get to make the decisions.” Edna said, “And I said ‘yes you do.’” At the same time, Edna pointed out that this is not what he would want. In this moment, Edna supported Patty’s right to make the decision while also encouraging her to consider what her son would want when that was a very difficult thing to face. Family members who were not considered primary decision makers acted as a support system to reinforce the decision that was made and to show support for the surrogate decision maker.

The decision-maker’s role enactment was undermined when family members engaged in conflict around who should take on the role or what the right decision should be. In Jackie’s situation, for example, family conflict emerged around whether or not the baby of the family should get to make decisions, even though she had legal standing as the surrogate decision maker. In contrast, Lynn, who was caring for a mother with dementia, indicated that her siblings were divided over the decision to keep her mother in her home, with four supporting the decision and three not supporting it. At one point, she said “we were one man down for a while, could you help us and all three of them said ‘if you need help, put her in a nursing home.’” Family members were not always consistently unsupportive in their response to the decision-maker’s behavior. Phyllis, for example, reported that her dad “was very argumentative with me and using a lot of foul language and kind of yelling at me” when she made the decision to decline a pacemaker for her mom. At other times, however, he had been supportive and encouraging given the difficult decisions she faced.

#### 3.2.2. Physicians and Medical Professionals and Role Enactment

Other family members were not the only ones to influence the enactment of the decision-maker role. Physicians and medical professionals played an important part in supporting the decision maker in his or her role or in supporting the family as a collective. In addition, physicians were an essential source of information for the decision-making process, and how they responded to questions also shaped the enactment of the decision-maker role.

For the most part, physicians respected multiple voices in the family decision and encouraged family meetings about the decision, regardless of whether or not a designated surrogate decision maker had been named. In other cases, physicians played a role in ensuring that the family as a decision-making unit was honored. For example, Molly reported that her mother was the formally designated decision maker when her father had a stroke. However, she indicated that the physicians engaged all of the family members in discussion about her father’s care at the end of life, helping the family to make a decision collectively. She said, “When the doctors talked to us, they looked at everybody…” In the end, all of the family members came together to play a part in the decision, which, according to Molly, was supported by the hospital staff. “Everybody got equal amount of respect from the doctors and the nurses, “she said. This support provided space for the kind of collective interaction that was important for families, regardless of the type of decision-making role being enacted. 

If, however, families engaged in conflict or hesitated in making a decision, the physician often encouraged the formal surrogate to step in to make the decision and reinforced the centrality of his/her legal position for taking on that kind of decision-making responsibility. Similarly, if families were not following the formalized wishes of the person at the end of life, physicians seemed to encourage those family members who were committed to following the formal wishes of the person at the end of life to take a lead. Jackie, for example, was faced with making a decision to withdraw treatment that was consistent with her mother’s wishes but conflicted with her siblings’ preferences. This conflict motivated the physician to encourage her, as the designated decision maker, to take the lead: “It came to uh… the point of the physician talking to me one on one. And telling me it was my duty and it was my responsibility…. He said, ‘This is not about them; this is about your mom.’ And he said, ‘You need to regain your focus.’”

In very few situations, participants also indicated that physicians engaged in behavior that undermined their ability to effectively perform the decision-making role. Most often, this revolved around failing to provide important information. Across interviews, it was clear that physicians provided key information that helped decision makers and family members understand the nature of the decision that they faced and orient to the possible consequences of different decisions. Questioning medical professionals was an important part of the decision-maker role. Molly, whose family reflected the collaborating pattern of role enactment, described everyone [in the family] asking questions “like a round table”. When physicians were ambiguous or avoided sharing difficult information, this made decision-making more difficult. Beatrice, for example, was frustrated with her father’s physician who did not provide important information for deciding whether or not to shift to hospice. Due to his reluctance to share difficult information, her persistent questioning of the doctor became a part of her enactment of the decision-maker role. 

## 4. Discussion

The primary decision-maker role is a key role in EOL decision-making [5]. This study offers insight into the expectations and behaviors that constitute that role in families when decisions must be made. The findings of this study highlight the importance of collective family interaction as a part of the decision-makers’ role enactment and the significance of family interaction for facilitating or inhibiting the enactment of the role. Additionally, this study provides insight into the ways in which the larger cultural, legal, and medical contexts intersect with specific family interaction to shape the structure and performance of the decision-maker role. 

### 4.1. Family Interaction and the Enactment of the Decision-Maker Role

Across decision situations, interaction with other family members was an important part of the role enactment. This is consistent with research indicating that families in the United States often prefer family conversations about decisions over individual decision-making [9,10,20]. The findings demonstrate, however, that there can be important variation in the nature of that family interaction in relation to the decision-making role. In some families, the collective input was a key component of the decision-making, and in other family contexts, collective conversations were an opportunity for the decision maker to help other family members understand what decision should be made. 

Although the U.S. legal system emphasizes the role of an individual surrogate decision maker, some families chose to construct the role as either a collaborative or consultative process rather than individual action. This occurred even in decision situations in which the patient had designated a specific person to be the legally recognized decision maker. For families who enacted a collective group decision-maker role or who had an individual decision maker who actively consulted other family members and adapted to their perspectives, collaborating and consulting created a more distinctive shared responsibility for the decision. Making an EOL decision for a family member carries a significant emotional burden [3,36], and diffusing the responsibility for the decision across family members may be one way to mitigate or share the emotional weight of the decision. 

Additionally, a desire to develop a shared understanding might help explain the informing approach to decision-maker role enactment. In those families, individual decision makers used the family’s collective interaction to explain the decision to other family members. In these cases, the surrogate decision maker’s responsibilities extended beyond making a decision for the patient to also ensuring that the family understood the rationale for the decision. Family interaction was oriented around educating other family members about what the patient would want, rather than jointly reaching a decision together.

Regardless of the type of role enactment, the construction of the decision-maker role as one that engages collective family input reflects the complexity of making a decision in the context of an on-going, interdependent relational system. As long as it is not conflict laden, family interaction in EOL decision-making may help to create a shared perspective on whether or not we are “doing the right thing”, something that family members struggle with as they face EOL decisions [3,24]. In addition, incorporating collective family interaction in the decision-making role, regardless of the form that it takes, may be one way in which families coordinate interaction so that they are able to go on together after a loved one has died. It is likely that what occurs in family interaction at the end of life, including the way in which the decision-maker role is enacted, has important consequences for how family members relate to each other after the death of their loved one. 

### 4.2. Enactment of the Decision-Maker Role within the Cultural, Legal, and Medical Context of the United States 

#### 4.2.1. Understanding the Patients’ Wishes and the Enactment of the Decision-Maker Role

Debate exists about whether or not family members are the best people to make decisions at the end of life. Whereas some scholars argue that their lack of medical expertise and/or emotional distress might undermine their decision-making capacity (e.g., [37]), others contend that they are uniquely positioned to know the values and preferences of the patient (e.g., [17]). Family members in this study, however, did not question whether or not a family member was the appropriate person to be making a decision for a loved one. The few conflicts that were reported oriented around the specific decision being made or who in the family was making the decision, but did not challenge the legitimacy of a family member as the appropriate decision maker. At the same time, however, the patients’ wishes were clearly essential to knowing how to be a good decision maker for many of the participants in this study and their family members. This family emphasis on what the patient would want reflects a cultural emphasis on autonomy and patient self-determination that has appeared in other EOL studies in the United States [3,19]. 

The importance of a person’s EOL wishes for families to know what to do as well as the significance of family support and collective interaction in decision-making point to the value of ensuring that family members other than the designated decision maker know one’s EOL preferences. Researchers have encouraged families to have informal discussions among family members about EOL preferences in addition to formal planning [29]. Findings from this study clearly support that need, given the influence of informal family discussions on decisions regardless of whether or not a formal decision maker was designated. Engaging family members, broadly defined, in collective discussions about patients’ EOL care needs, ensures that all involved parties are more likely to understand and respect patients’ preferences. 

#### 4.2.2. Medical Professionals and the Enactment of the Decision-Maker Role

Based upon the pattern of findings in this study, medical professionals helped to bolster and support decision makers and reinforce the legitimacy and importance of that role to the family. Past research in the U.S. indicates that medical professionals can find family involvement in decision-making to be problematic, particularly when multiple different family members expect to participate in decision-making interaction [38]. Group interaction contradicts a medical model that emphasizes the relationship between the physician and the patient and/or a single surrogate decision maker [20,39]. Family members in this study, however, described ways in which physicians’ behaviors helped to support the inclusion of collective family input in the performance of the decision-maker role. Research has demonstrated that physicians and other medical professionals serve as an important source of information for families facing a difficult decision [21]. In serving as a source of information for families, physicians are in a position to support collective family discussions about EOL decisions through their communication with the family as a whole during this time. When family members experience conflict around who should be making EOL decisions, physicians also offer support for surrogate decision makers, reinforcing the legitimacy of their role as a decision maker.

#### 4.2.3. Family Roles, Age, Gender and the Decision-Maker Role

The decision-making role was clearly enacted within a familial context that carries with it a hierarchy of influence and responsibility in relationship to decision-making. Family, age and gender roles all appeared in family members’ explanation for who took on the decision-making role in the family. This hierarchy of influence is culturally grounded. Research, for example, indicates variation across ethnic and racial groups in the U.S. in what individuals identify as the preferred formal family role (e.g., spouse, child, sibling) to draw on for a surrogate decision maker [18]. In situations where the person taking on the decision-making role in this study did not fit cultural or legal expectations for who should be making a decision (e.g., an adult child instead of a spouse), participants offered explanations for why unexpected role performances occurred. 

## 5. Conclusions

The findings of this work offer valuable insight regarding how family members enact the decision-making role in family interactions surrounding the end-of-life decisions. However, the study is limited in several ways. First, the sample of this study was partially drawn from snowball methods, which limits the perspectives included. In contrast to other research interviewing surrogate decision makers (e.g., [40]), our participants had generally positive experiences with medical professionals and reported very little conflict with physicians. The influence of the physician in role enactment would be very different in contexts of disagreement around treatment decisions. Additionally, no participants who volunteered for the study reported on EOL decision-making for a spouse. Given the importance of the spousal role for EOL decision-making, the absence of the spousal perspective limits the transferability of findings. 

Future research should attend to both cultural and familial factors that shape family interaction during EOL decision-making. The intersection of autonomy and interdependence that emerges in work attending to family processes in EOL decision-making [39] reflects a particular cultural understanding of families and their relationship to EOL decisions. Cross-cultural comparisons regarding family structure and expectations [41] as well as beliefs about decision-making responsibility [42] would offer additional insight into family processes related to EOL decision-making. The relational processes and expectations that family members bring to the decision-making situation also likely shape the patterns that emerge in the family [41]. Molly, for example, suggested that the cohesiveness that characterized relationships within her immediate family network helped to explain why everyone was involved actively in making the decision together. Future research should explore the ways in which the relational dynamics that the family brings to the decision-making situation shape the roles that emerge.

The end of life of a family member is an emotionally challenging and complex site of family decision-making. How family members socially construct the decision-maker role is critical for the decisions that are made and the coordination of family members in the decision-making process. Given the importance of collective interaction for role enactment and the significance of family members’ responses for supporting or undermining that role enactment in EOL decisions, researchers and practitioners need to attend carefully to the interdependent family context when considering the process of EOL decision-making.
